# Effects of Individual Differences in Working Memory on Plan Presentational Choices

**DOI:** 10.3389/fpsyg.2016.01793

**Published:** 2016-11-16

**Authors:** Nava Tintarev, Judith Masthoff

**Affiliations:** ^1^Department of Computing and Informatics, Faculty of Science and Technology, Bournemouth UniversityPoole, UK; ^2^Department of Computing Science, School of Natural and Computing Sciences, Aberdeen UniversityAberdeen, UK

**Keywords:** visualization, evaluation, task interleaving, aggregation, visual working memory, plan presentation

## Abstract

This paper addresses research questions that are central to the area of visualization interfaces for decision support: (RQ1) whether individual user differences in working memory should be considered when choosing how to present visualizations; (RQ2) how to present the visualization to support effective decision making and processing; and (RQ3) how to evaluate the effectiveness of presentational choices. These questions are addressed in the context of presenting plans, or sequences of actions, to users. The experiments are conducted in several domains, and the findings are relevant to applications such as semi-autonomous systems in logistics. That is, scenarios that require the attention of humans who are likely to be interrupted, and require good performance but are not time critical. Following a literature review of different types of individual differences in users that have been found to affect the effectiveness of presentational choices, we consider specifically the influence of individuals' working memory (RQ1). The review also considers metrics used to evaluate presentational choices, and types of presentational choices considered. As for presentational choices (RQ2), we consider a number of variants including interactivity, aggregation, layout, and emphasis. Finally, to evaluate the effectiveness of plan presentational choices (RQ3) we adopt a layered-evaluation approach and measure performance in a dual task paradigm, involving both task interleaving and evaluation of situational awareness. This novel methodology for evaluating visualizations is employed in a series of experiments investigating presentational choices for a plan. A key finding is that emphasizing steps (by highlighting borders) can improve effectiveness on a primary task, but only when controlling for individual variation in working memory.

## 1. Introduction

An *autonomous system* consists of physical or virtual systems that can perform tasks without continuous human guidance. Autonomous systems are becoming increasingly ubiquitous, ranging from unmanned vehicles, to virtual agents which process information on the internet. Such systems can potentially replace humans in a variety of tasks which can be dangerous (such as refuelling a nuclear reactor), mundane (such as crop picking), or require superhuman precision (as in robotic surgery).

In practice, most of these systems are still *semi-*autonomous in the sense that they need human approval for execution, or can be interrupted by human operators (e.g., current commercial self-driving cars require a person to keep their hands on the wheel at all times). For these semi-autonomous systems to operate optimally, it is vital that humans understand the actions the system is planning. One way the system can communicate plans is visually. For this reason, this paper focuses on visual representations of *plans*.

When we talk about plans in this paper, we mean a suggested sequence and ordering of actions, that if followed will lead to a goal. Formally, these can be seen as states, and transitions which lead from a start state to a goal state (Ghallab et al., 2004). Often these plans are very difficult for humans to understand. In fact, for plans that are inherently complex, it can be sufficiently difficult to understand the information that they represent, let alone understand the justification behind the way the plan is constructed.

Plans can vary in terms of their *inherent* complexity (analogous to intrinsic cognitive load, Sweller, 1988), as well as their *presentation* complexity (analogous to extraneous cognitive load, Sweller, 1988). Examples of factors influencing presentation complexity include modality, layout, and degree of interactivity. Unlike inherent complexity, a designer of a system can control presentational complexity. Therefore, it is important to conduct studies to understand which presentational choices are less complex and place less cognitive load on users. Studies may also help identify whether presentational choices are effective for some users, but not others.

There may be choices that system designers can apply to reduce the presentational complexity of plans that are shown to human operators. With this in mind, this paper addresses core research questions in the area of visual presentational choices, in the context of presenting plans, or sequences of actions, to users.

Section 2 discusses literature in the area of information presentational choices for visualizations. It addresses which individual user differences should be considered that can support effective decision making and processing, since differences in users have been found to affect how people interpret visual information. This paper focuses specifically on one of the cognitive traits that has been found to affect how individuals interpret information, namely differences in working memory (e.g., digit span, visual memory) (RQ1).

The literature review also proposes metrics for evaluation and shows how visual presentational choices have been evaluated in the past. It helps us understand how to addresses RQ3: how to evaluate the effectiveness of presentational choices. Many real-world applications of plans are likely to involve people interrupting one task to handle another, potentially returning to the original task thereafter. So, the review will particularly highlight research in this area of *task interleaving*. Following this review, we introduce a methodology for evaluating performance during task interleaving (Section 3).

The literature review also describes information presentational choices for visualizations (RQ2). We follow with 4 experiments (Sections 4–7) that use the proposed methodology to evaluate how different presentational choices (interactivity, aggregation, layout, and emphasis) influence task performance. We conclude with implications and suggestions for future work in Section 8.

## 2. Related work

This section discusses related work addressing how visual presentational choices have been applied and evaluated in the past.

### 2.1. RQ1: whether individual user differences in working memory should be considered

Anecdotal evidence about individual differences has motivated research on presenting the same information in different visualization views (Wang Baldonado et al., 2000). While our work looks specifically at working memory, we contextualize our choice with findings with measurable variation between individuals based on a number of factors such as cognitive abilities (Velez et al., 2005; Toker et al., 2012) (including working memory), personality (Ziemkiewicz et al., 2011), and degree of expertise or knowledge (Lewandowsky and Spence, 1989; Kobsa, 2001). In addition, gender and culture may be factors to consider for visual presentational choices given known gender differences in processing spatial information, and cultural differences in spatial density of information (Hubona, 2004; Velez et al., 2005; Fraternali and Tisi, 2008).

#### 2.1.1. Personality

Studies have evaluated whether personality traits affect individuals' abilities to interpret visualizations. In trait theory, a trait is defined as “an enduring personal characteristic that reveals itself in a particular pattern of behavior in different situations” (Carlson et al., 2004, p. 583). One study found an interaction of the personality trait of *locus of control*, with the ability to understand nested visualizations (Ziemkiewicz et al., 2011).

Another study evaluated the general effect of personality on completion times, and number of insights, but did not study the interaction between information presentational choices and personality (Green and Fisher, 2010). This study also looked at locus of control, and two of the “Big Five” personality traits: *Extraversion* and *Neuroticism*. Participants who had an intrinsic locus of control, or scored higher on the traits of extroversion and neuroticism were found to complete tasks faster. In contrast, participants who had an external locus of control, or scored lower on Extraversion and Neuroticism gained more insights.

#### 2.1.2. Expertise

Another trait that has been considered is the level of expertise of the user. Küpper and Kobsa Kobsa (2001) proposed adapting plan presentation to a model of a user's *knowledge and capabilities* with regard to plan concepts, e.g., knowledge of the steps and the relationships with them. Others have formally evaluated the effect of familiarity of the data presented and individuals' graphical literacy on abilities to make inferences (from both bar and line charts) (Shah and Freedman, 2011). Individual expertise in using each particular type of graphs (radar graphs and bar graphs) also influenced the usability of each respective type of graph (Toker et al., 2012).

#### 2.1.3. Cognitive traits

The influence of individual cognitive traits has been shown consistently to influence the understandability of visualizations. Studies have also considered a number of related cognitive abilities. Previous studies have found significant effects of cognitive factors such as *perceptual speed, verbal working memory, visual working memory* on task performance (Toker et al., 2012; Conati et al., 2014). Other studies have also found an effect of individual perceptual speed, and visual working memory capacity, on which visualizations were most effective (Velez et al., 2005; Conati et al., 2014). For example, participants with low visual working memory were found to perform better with a horizontal layout (Conati et al., 2014). These findings suggest that cognitive traits are particularly promising factors to personalize presentational choices to in domains with high cognitive load. We further motivate the trait we chose to study, *working memory*, in Section 3.

### 2.2. RQ2: how to present the presentation

This section describes some of the choices that can be made about how to present a plan: modality, layout, degree of interactivity, aggregation and emphasis.

#### 2.2.1. Modality

**Figure 1:**
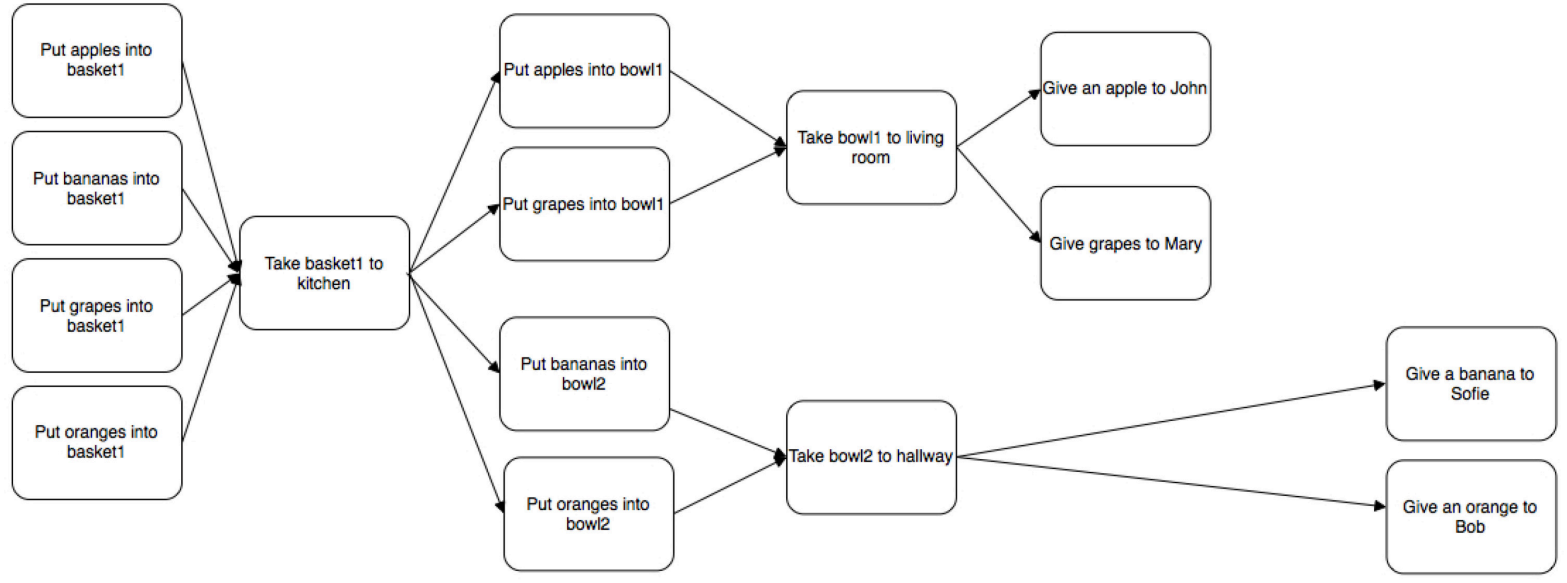
**Example of a simple plan visualization**.

Plans can be presented in textual form (Mellish and Evans, 1989; Biundo et al., 2011; Bercher et al., 2014), and as visualizations (Küpper and Kobsa, 2003; Butler et al., 2007; Brown and Paik, 2009; McCurdy, 2009; Billman et al., 2011; de Leoni et al., 2012) (with some variants somewhere on a continuum). Given that users are variable in terms of their verbal working memory (Toker et al., 2012; Conati et al., 2014), the choice of modality is a candidate for design choice. Figure 1 shows a simple plan visualization where nodes describe actions, and edges are transitions to other actions.

#### 2.2.2. Layout

The way plans are laid out can help or hinder their presentational complexity. For example, the visual layout can use a mapping most suitable for that domain. The mapping used has differed between different planning domains, for example mapping to a location resource in the domain of logistics (de Leoni et al., 2012), or horizontal alignment according to time for tasks that are constrained by time. For example, Conati et al. (2014) found that users with low visual working memory answer more answers correctly with a horizontal layout compared to a vertical layout for complex visualizations (ValueCharts). Other work has compared the same information presented as a bar chart vs. a radar graph (Toker et al., 2012).

#### 2.2.3. Degrees of interactivity

As plans get large it may be necessary to occlude parts of a plan to support an overview. The idea of fading (Hothi et al., 2000) and hiding parts (e.g., using stretchtext Boyle and Encarnacion, 1994) of information presentation (primarly text) has previously been explored in the area of hypertext. Research on stretchtext has investigated the effectiveness of choosing which information is shown (i.e., “stretched”) and which is not (but available, via selection, i.e., “shrunk”). In the area of graphs, Henry (1992) looked at filtering graphs by content, and Sarkar and Brown (1992) applied fish-eyes views to grow or shrink parts of a graph. Other work has supported zooming to manage the visualization of larger plans (Billman et al., 2011; de Leoni et al., 2012).

#### 2.2.4. Aggregation

By aggregation, we mean gathering of several things together into one thing. For example, the making of dough can include several composite steps such as adding flour or mixing the ingredients, but it can also be aggregated into a single step of “making dough.” In other words, an alternative method for dealing with large plans is to support the cognitive mechanism of chunking, by representing several steps by higher order concepts. For example, Eugenio et al. (2005) found that describing instructions in natural language using aggregated concepts (such as the concept of an engine rather than listing all of its composite parts) lead to greater learning outcomes. This method can also be combined with interactivity using methods such as stretchtext (mentioned above) to contract or expand relevant portions of a plan. Aggregation would also benefit from considering a user's expertise or experience with a particular task (Kobsa, 2001). Several of the surveyed planning systems support concepts similar to main task and sub-tasks, where a main task can consist of several sub-tasks, c.f., Billman et al. (2011). In contrast, several levels of aggregation could make presentation more complex, e.g., Gruhn and Laue (2007) claims that a greater nesting depth in a model increases its complexity. Users have also been found to differ on how well they perform tasks using visualizations with nesting or aggregation; users who scored low on the personality trait of internal locus of control performed worse with nested visualizations when comparing with users who scored highly on the trait (Ziemkiewicz et al., 2011).

#### 2.2.5. Emphasis

Both text and graphics can be visually annotated to indicate importance, for example by changing their size or color to indicate relevance (Brusilovsky et al., 1996). Conversely, dimming and hiding has been used to de-emphasize information (Brusilovsky et al., 1996). Color is a particularly good candidate for emphasis; work in visual processing has established that color is processed much quicker by the visual system compared to other highly salient visual features such as shapes (Nowell et al., 2002). This fact has implicitly been taken into consideration in interactive learning environments (Freyne et al., 2007; Jae-Kyung et al., 2008). Color highlighting specifically is a recognized technique for adapting *hypertext and hypermedia* (Brusilovsky et al., 1996; Bra et al., 1999; Jae-Kyung et al., 2008; Knutlov et al., 2009), and is possibly the most commonly used type of emphasis (Butler et al., 2007; Brown and Paik, 2009; McCurdy, 2009; Billman et al., 2011; de Leoni et al., 2012), however systems also used other visual encodings to distinguish between different types of information such as *relative differences* in sizes and shapes (c.f., de Leoni et al., 2012).

### 2.3. RQ3: how to evaluate the effectiveness of presentational choices

The aims of visualization evaluations have varied (Lam et al., 2012). First of all, it is worth to distinguish between *what* one is evaluating (e.g., data abstraction design vs. presentational encoding), and *how* (e.g., lab studies, ethnographic studies) one is evaluating it (Munzner, 2009). We also follow most closely the sort of evaluations that could be classed under Lam et al's header of “evaluating human performance” that study the effects of an interactive or visual aspect of the tool on people in isolation.

To enable this we supply an overview of previously applied evaluation criteria, broaden our view from visualizations to include earlier work on information presented as hypertext or hypermedia.

#### 2.3.1. Efficiency

Broadly speaking, efficiency can be defined as “Helping users to perform their tasks faster.” In previous studies efficiency has been measured as time to complete a single task, a set of tasks, or the number of tasks per hour (Campagnoni and Ehrlich, 1989; Egan et al., 1989; McDonald and Stevenson, 1996). Alternative measures, such as the number or types of interactions have also been used (Kay and Lum, 2004). Efficiency can be affected by the choice of visualization, but also depends on the task and user characteristics (Toker et al., 2012). For example, previous work found that perceptual speed influenced task completion times using bar chars and radar charts. In addition, they found an interaction between visualization type and perceptual speed: the difference in *time performance* between bar and radar charts decreases as a users' perceptual speed increases. A similar study evaluating a more complex visualization called ValueCharts measured the interaction between task type and various cognitive traits (Conati et al., 2014). They found that level of user expertise, verbal working memory, visual working memory, and perceptual speed all interacted with task type (high level or low level). That is, the influence of individual traits on speed of performance depended on the type of task that was being performed as well. Another study measured the effect of the personality trait of locus of control on the *time spent on correct responses* (Ziemkiewicz et al., 2011). Overall, participants who scored low on locus on control were slower at answering questions correctly. There was also an interaction with the trait and question type, for some questions (search tasks) participants who scored low on the trait were as fast as participants who scored highly.

#### 2.3.2. Effectiveness

In the most general sense, a system can be said to be effective if it helps a user to produce a desired outcome, i.e., “Helps users to perform their tasks well.” The nature of the tasks naturally varies from system to system. For example, in a decision support context, such as recommender systems, effectiveness has been defined as “Help users make good decisions” with regards to whether to try or buy an item (Tintarev and Masthoff, 2015). For plans, this means to successfully complete a task which requires several actions. This task may require completing the actions originally suggested by the system, or a revised sequence resulting from a user scrutinizing and modifying the suggested plan.

The most common method for evaluating information visualizations' effectiveness has been to ask participants to answer questions based on the information presented (Campagnoni and Ehrlich, 1989), although this could be said to measure understandability rather than effectiveness (Section 2.3.7). For example, Conati et al. (2014) found that users with low visual working memory answer more answers correctly with a horizontal layout (compared to a vertical layout). The complexity of the questions has varied from simple tasks (e.g., searching for objects with a given property, specifying attributes of an object or performing count tasks; Stasko et al., 2000; Conati et al., 2014), to more complex ones (e.g., questions covering three or more attributes; Kobsa, 2001; Verbert et al., 2013). Consequently, studies of both visualization (Stasko et al., 2000; Kobsa, 2001; Indratmo and Gutwin, 2008), and hypertext (Campagnoni and Ehrlich, 1989; Chen and Rada, 1996), have been evaluated in terms of *error rates or correct responses*. Effectiveness has also been measured as *how frequently a task was successful*, for a task such as bookmarking at least one interesting item (Verbert et al., 2013). Other measures such as *coverage* (proportion of the material visited) have also been used (Egan et al., 1989; Instone et al., 1993).

#### 2.3.3. Perceived measures of effectiveness and efficiency vs. actual measures

One way to supplement measures of effectiveness and efficiency is to ask participants to self-report. Self-reported measures have been found to be reliable and sensitive to small differences in cognitive load in some cases (Paas et al., 2003). That is, if participants perform better but take longer, a self-reported measure of high mental effort could confirm that cognitive load was high. There are justified questions about the reliability of self-reported measures as well, for example, people are known to be worse at correctly judging the time a task takes when under heavy cognitive load (Block et al., 2010). This suggests that self-reported measures may be a good supplement, but not a replacement for the actual measures. Visualizations have also been evaluated in terms of perceived *effort* when performing a task (Waldner and Vassileva, 2014). One commonly used measure for *subjective workload* is the NASA TLX, which uses six rating scales for: mental and physical demands, performance, temporal demand, effort and frustration (Hart, 2006).

#### 2.3.4. Task resumption lag

One criteria that is underrepresented in evaluations of visualizations is task resumption lag. This metric is particularly relevant in real world applications where interruptions are likely, and where the user is likely to be under heavy cognitive load. Task interleaving is a phenomena that happens in many multi-tasking applications, and is not limited to the evaluation of plans. This interleaving takes time, and poses a cognitive effort on users. One concrete way the cost of task interleaving has been evaluated is the time it takes to resume a previously interrupted task, or resumption lag (Iqbal and Bailey, 2006). Previous studies have identified a number of factors that influence resumption lag, including how frequent (or repeated) interruptions are, task representation complexity, whether the primary task is visible, whether interleaving happens at task boundaries, and how similar concurrent tasks are to each other (Salvucci et al., 2009).

Some see task interleaving as a continuum from few interruptions, to concurrent multi-tasking (where joint attention is required) (Salvucci et al., 2009). A dual-task paradigm is a procedure in experimental psychology that requires an individual to perform two tasks simultaneously, to compare performance with single-task conditions (Knowles, 1963; Turner and Engle, 1989); (Damos, 1991, p. 221). When performance scores on one and/or both tasks are lower when they are done simultaneously compared to separately, these two tasks interfere with each other, and it is assumed that both tasks compete for the same class of information processing resources in the brain. Examples of where it may be important to measure task resumption lag include piloting, driving, and radar operation. For example, a pilot executing a procedure may be interrupted by the control center.

A dual task methodology has been previously used to evaluate information visualization. Visual embellishments (e.g., icons and graphics) in terms of memorability (both short and long term memory), search efficiency, and concept grasping (Borgo et al., 2012).

#### 2.3.5. Satisfaction

Satisfaction gives a measure of how much users like a system or its output (i.e., the presented plans). The most common approach used is a questionnaire evaluating subjective user perceptions on a numeric scale, such as how participants perceived a system (Bercher et al., 2014). It is also possible to focus on satisfaction with specific aspects of the interface such as how relevant users find different functionalities (Apted et al., 2003; Bakalov et al., 2010), and how well each functionality was implemented (Bakalov et al., 2010). A variant is to compare satisfaction with different variants or views of a system (Mabbott and Bull, 2004). Individual expertise in using particular types of graphs (radar graphs and bar graphs) has been found to influence preference for which type of graph people perceived as easier to use (Toker et al., 2012).

#### 2.3.6. Memorability

Memorability is the extent to which somebody can remember a plan. This can be tested through recall (can the user reconstruct the plan) and through recognition (can the user recognize which plan is the one that they saw previously). Dixon et al (Dixon et al., 1988, 1993) showed that the memorability of plans can be affected by the representations used (in their case the sentence forms used). Kliegel et al. (2000) found that participants' working memory and plan complexity influence plan memorability (which they called plan retention) and plan execution. Recall of section headers (as a measure of incidental learning) have also been used (Hendry et al., 1990). In measuring memorability, it may be important to filter participants on general memory ability, e.g., excluding participants with exceptionally good or poor memory. In domains where users repeat a task it may also be valuable to measure performance after a certain training period as performance on memorability has been found to stabilize after training (Schmidt and Bjork, 1992). Measurements of memorability are likely to show improved performance with rehearsal. Previous evaluations of information visualizations have considered both short term and and long term memorability (Borgo et al., 2012).

#### 2.3.7. Understandability

Understandability (also known as ComprehensibilityBateman et al. (2010)) is the extent to which the presented information is understood by participants. Understandability of information can be measured by asking people to summarize its contents (Bloom et al., 1956), answer questions about its contents (called Correctness of Understanding by Aranda et al., 2007), or by using a subjective self-reporting measure of how easy it is to understand (Hunter et al., 2012). For the latter, a distinction is sometimes made between *confidence*, the subjective confidence people display regarding their own understanding of the representation, and *perceived difficulty*, the subjective judgement of people regarding the ease to obtain information through the representation (Aranda et al., 2007). Biundo et al. (2011) and Bercher et al. (2014) evaluated a natural language presentation and explanation of a plan. The evaluation task was to connect multiple home appliances. The main evaluation criteria was primarily perceived certainty of correct completion (confidence aspect of understandability), but they also measured overall perceptions of the system (satisfaction). A study evaluating perceived ease-of-use found an effect of verbal working memory on ease-of-use for bar charts (Toker et al., 2012).

As plan complexity impacts understandability, there is also research on measuring understandability by analysing this complexity, for example in business process models (Ghani et al., 2008) and workflows (Pfaff et al., 2014). Aranda et al. (2007)'s framework for the empirical evaluation of model comprehensibility highlights four variables that affect comprehensibility, namely language expertise (previous expertise with the notation/representation being studied), domain expertise (previous expertise with the domain being modeled), problem size (the size of the domain), and the type of task (for example, whether readers need to search for information, or integrate information in their mental model). One of the tasks mentioned by Aranda et al. (2007), namely information retention, is covered by our Memorability metric.

#### 2.3.8. Situational awareness

Situational awareness is the users' perception of environmental elements with respect to time and space, the comprehension of their meaning, and the projection of their status after some variable has changed (Endsley and Jones., 2012). It is often classified on three levels (Endsley and Jones., 2012): Level 1—the ability to correctly perceive information; Level 2—the ability to comprehend the situation, and Level 3—projecting the situation into the future. Abilities to make decisions in complex, dynamic areas are therefore concerned with errors in situational awareness. In particular, Level 3 expands the situational awareness beyond the regular scope of understandability (c.f., Section 2.3.7). Adagha et al. (2015) makes a case that standard usability metrics are inadequate for evaluating the effectiveness of visual analytics tools. In a systematic review of 470 papers on decision support in visual analytics they identify attributes of visual analytics tools, and how they were evaluated. Their findings imply a limited emphasis on the incorporation of Situational Awareness as a key attribute in the design of visual analytics decision support tools, in particular with regard to supporting future scenario projections. Situational awareness is strongly linked to what Borgo et al. (2012) call *concept grasping* and define as: “more complex cognitive processes of information gathering, concept understanding and semantic reasoning.”

#### 2.3.9. Trade-offs between metrics

The metrics mentioned above provide a useful way of thinking about ways of evaluating visualizations. However, it is unlikely that any choices about how to present a plan will improve performance on *all* these metrics. For example, effectiveness and efficiency do not always correlate. For example, high spatial ability has been found to be correlated with accuracy on three-dimensional visualization tasks, but not with time (Velez et al., 2005). One method that has been used is to record the time for successful trials only (Ziemkiewicz et al., 2011). Similarly, a meta-review of effectiveness and efficiency in hypertext found that the overall performance of hypertext users tended to be *more effective* than that of non-hypertext users, but that hypertext users were also *less efficient* than non-hypertext users (Chen and Rada, 1996). This is also reflected in the literature in psychology where a single combined measure of effectiveness and efficiency has been found to have very limited use (Bruyer and Brysbaert, 2011).

Another useful distinction is between memorability at first exposure, and long term effectiveness. In some applications, it may be important for a user to quickly learn and remember the contents and plans. In others, the task may repeat many times and it is more important that effectiveness stabilizes at an acceptable level after a degree of training.

Two other metrics that are known to conflict with regard to information presentation are effectiveness and satisfaction. For example, in one study, while participants subjectively preferred a visual representation (Satisfaction), they made better decisions (Effectiveness) using a textual representation of the same information (Law et al., 2005).

## 3. Overview and methodology of experiments

This section describes a general methodology applied in three out of the four experiments conducted. The experiments contribute to answering the three research questions: (RQ1) whether individual user differences in working memory should be considered when choosing how to present visualizations; (RQ2) how to present the visualization to support effective decision making and processing; and (RQ3) how to evaluate the effectiveness of presentational choices.

We consider the influence of participants' working memory on the effectiveness of decision-making for different presentational choices (**RQ1**). We chose to consider a cognitive trait that is likely to be important in domains with high cognitive load, which may amplify individual differences in performance. While previous studies have found significant effects for perceptual speed, this affected primarily task duration—a factor less relevant in domains that are not time critical. Rather, our work looks specifically at working memory, which has been shown to interact with presentational choices, such as layout, in particular. In our experiments, we consider two kinds of working memory: a digit span task, and visual working memory (a Corsi test, as in Conati et al., 2014).

Above, we mentioned that presentational complexity could influence the “goodness” of a plan (independent of its inherent complexity, such as size). Therefore, to address **RQ2** we will modify different aspects of plan presentation to evaluate their influence on effectiveness. To assess the benefit of each presentational choice (**RQ3**), we apply a dual-task paradigm often used in cognitive psychology (Turner and Engle, 1989). This allowed us to evaluate the interaction between working memory (individual trait) and performance. We describe this methodology further in Section 3.1.

Table 1 summarizes the experiments, and the presentational complexity factors considered. The four studies are conducted in multiple domains. We also considered several ways of modifying the presentation of the plans: modality, layout, interaction, aggregation and emphasis. Two experiments were conducted using Amazon Mechanical Turk, and two were more controlled studies in the lab. We note that crowd-sourced graphical perceptions have been found to replicate the results from prior lab-based experiments, while decreasing cost and increasing completion rates (Heer and Bostock, 2010).

**Table 1:** **The domains, and presentational choices, applied in the experiments**.

	**Domain**	**Modality**	**Layout**	**Interact**.	**Aggreg**.	**Emphasis**
Exp1, mTurk	Logistics	Text	Tree	Expand on +	Expand on +	−
Exp2, mTurk	Logistics	Graph with labels	Horizontal (by location and sequence)	Button selection	−	Solid border and pre-condition (color)
Exp3, Lab	Various	Graph with labels	Horizontal (by location and sequence)	−	−	Solid blue border
Exp4, Lab	Logistics	Graph with labels	Horizontal (by location)	Button selection	−	Solid blue border, and dotted for pre-conditions

Slight variations in each experiment are described for each experiment in Sections 4–7. Experiment 3 used a different methodology, which is reported and justified in Section 5.

### 3.1. Experimental design

At the core of the general methodology is the dual-task paradigm often used in cognitive psychology (Knowles, 1963; Turner and Engle, 1989); (Damos, 1991, p. 221). That is, we measured the performance on a primary task, while under cognitive load from a secondary task. The justification for applying this method is that tasks performed in the real world are likely to be competing with other distracting tasks in parallel, and it is vital in domains where task interleaving occurs to be aware of the impairment on both the main and secondary tasks. More specifically, it also has the advantage of simulating task interleaving (Gross et al., 2006; Iqbal and Bailey, 2006; Salvucci et al., 2009).

In addition to the dual-task setting, our experimental design considers *individual variation* in memory, as this is likely to impact on performance in a dual task, and is known to impact the effectiveness and efficiency of information visualization (Chen and Yu, 2000; Conati et al., 2014). We used a between-subject design.

We apply a layered evaluation approach used in adaptive interactive systems for evaluating specifically the information presentational choices (Paramythis et al., 2010). We note that this is compatible with the frame framework for visualization suggested by Munzner which includes four levels: Problem characterization; Data/operation abstraction design; Encoding/interaction technique design; Algorithmic design (Munzner, 2009). In this paper, we primarily consider the evaluation of the layer of what Munzner calls the Encoding/interaction technique design, building on the abstractions and problem characterizations commonly found in AI planning and logistics respectively. For example Experiments 2–4 considered a layout which describes a logical ordering (left-to-right), and geographic location (vertical “lanes”) that is influenced by the norms in the logistics domain. We do not evaluate the fourth layer of algorithmic design or efficiency.

### 3.2. Hypotheses

In our experiments we evaluate several types of presentational choices (addressing RQ2, how to present the visualization). We also address the issue of how to evaluate effectiveness (RQ3), by applying the dual task methodology described above. Experiments 1, 2, and 4, have the same first two hypotheses:

**H1.** The number of correct answers in the secondary task will differ significantly between the representations.
**H2.** The number of correct answers in the primary task will differ significantly between the representations.

### 3.3. Participants

Participants were recruited from Amazon Mechanical Turk (mTurk), and were paid $1.50 USD for an estimated completion time of 15 min. We required participants to both be based in the US and have a minimum approval rating of 90%. We recorded the unique worker IDs of participants who completed the experiment to avoid repeat participation. This also means that participants across experiments are distinct.

In addition, to ensure a high quality of responses (and exclude automated or spurious responses) the experiment early on excluded participants who had below average memory, with a digit span of 4 or lower^1^. An exception to this design are the lab studies, and differences are outlined in Section 5 and Section 7.

### 3.4. Procedure

The procedure contained the following steps:

1. *Measurement of participant memory* - We used either a memory digit span task (Blankenship, 1938) (in Sections 4 and 6), or a visual memory test (Corsi block span Corsi, 1972, in Section 7). In the memory span task, a random number was presented one digit at a time with each digit shown for one second. After the last digit, participants recalled the number. The test began with a number with two digits, increasing the number of digits until the person committed two errors for the same length or until length 9 was reached. The visual test highlights squares in a screen, one at a time. Participants need to select the squares in the order they were highlighted. The number of squares increased until the person got 2 out of 2 sequences of a given length wrong. The memory test was also used as quality control (both in terms of participant motivation, and the effect of poor memory on the main task); participants who performed below average were removed from analysis.
2. *Secondary task.* The secondary task across all four experiments was a serial recall of a list of words. This task plays the roles of (a) increasing cognitive load and (b) simulating task interleaving in the real world. Serial recall was selected as it is a distinct task from the primary task, and places demands on a different type of working memory (more verbal than visual). Participants had a limited amount of time to remember as many words as possible from a list. While the list disappeared from view after the allotted time, participants had control over the task interleaving, and themselves determined when they went on to the next task.
3. We used a wordlist of size 8 to avoid a ceiling effect in recall rates across conditions: sum, hate, harm, wit, bond, yield, worst, twice. This list was used in previous recall tasks, which found that less than 20% of the participants remembered the series correctly (Baddeley et al., 1975). Participants were given 20 s remember this list.
4. *Primary task.* The primary task involves answering questions, of varying problem representation complexity, about a plan. Participants were able to view and/or interact with the plan while answering these questions, and thus had access to any visual cues that might help them with the task.
5. *Secondary task continued.* After the primary task was completed the participants returned to the secondary task. They were asked to write down as many of the items from the word list they could recall, in the correct order.

### 3.5. Independent measures

In each experiment, we varied the way the plan was represented, and compared the representational choices between subjects. While the materials between experiments varied, the same material was used within each experimental setting.

### 3.6. Dependent measures

Each experiment measured: (a) Effectiveness on primary task–the number of correctly answered questions, and (b) Effectiveness on secondary task–the number of correctly remembered words, in the right position.

## 4. Experiment 1: interactivity and aggregation (mTurk)

This experiment studied the effects of interactivity (2 levels: interactive/static) and aggregation (2 levels: aggregated/regular), in textual plans. By aggregation we mean that labels were given for sequences of steps (summarizing 3 steps), and that we allowed for several levels of nesting. The aggregation also allowed investigating the effects of interactivity in plan presentations. These two presentational choices (interactivity and aggregation) are two alternatives that we investigated as part of RQ2 (how to present the plan visualization). We consider how these interact with working memory (RQ1), and apply a dual-task methodology to evaluate the presentational choice (RQ3).

### 4.1. Procedure

The experiment ran on mTurk, and followed the procedure outlined in Section 3. We disabled the ctrl/meta-F button which triggers the browser's search function. If participants used the search function, this could improve the performance and efficiency of answering questions about the plan in the static condition. The intention was to block the quickest avenue toward search (key combination). Participants who made a larger effort to search could still use this functionality from the main browser menu however.

### 4.2. Participants

Sixty-six participants were included in the analysis. Fifteen participants were excluded for durations lower than 8 min (more than 2 SD away from the mean), 6 for mean memory less than 5. Participants self-reported to be nearly evenly divided between genders (41.0% female, and 59.1% male). The majority (57.6%) of participants self-reported to be aged 26–50, 27.3% were younger, and 15.2% were older. The mean digit span by participants was 7.53 (1.15), and the mean time to complete the plan questions was 12 min 14 s (SD 2 min 1 s).

### 4.3. Materials

#### 4.3.1. Presentational choices for plans

**Figure 2:**
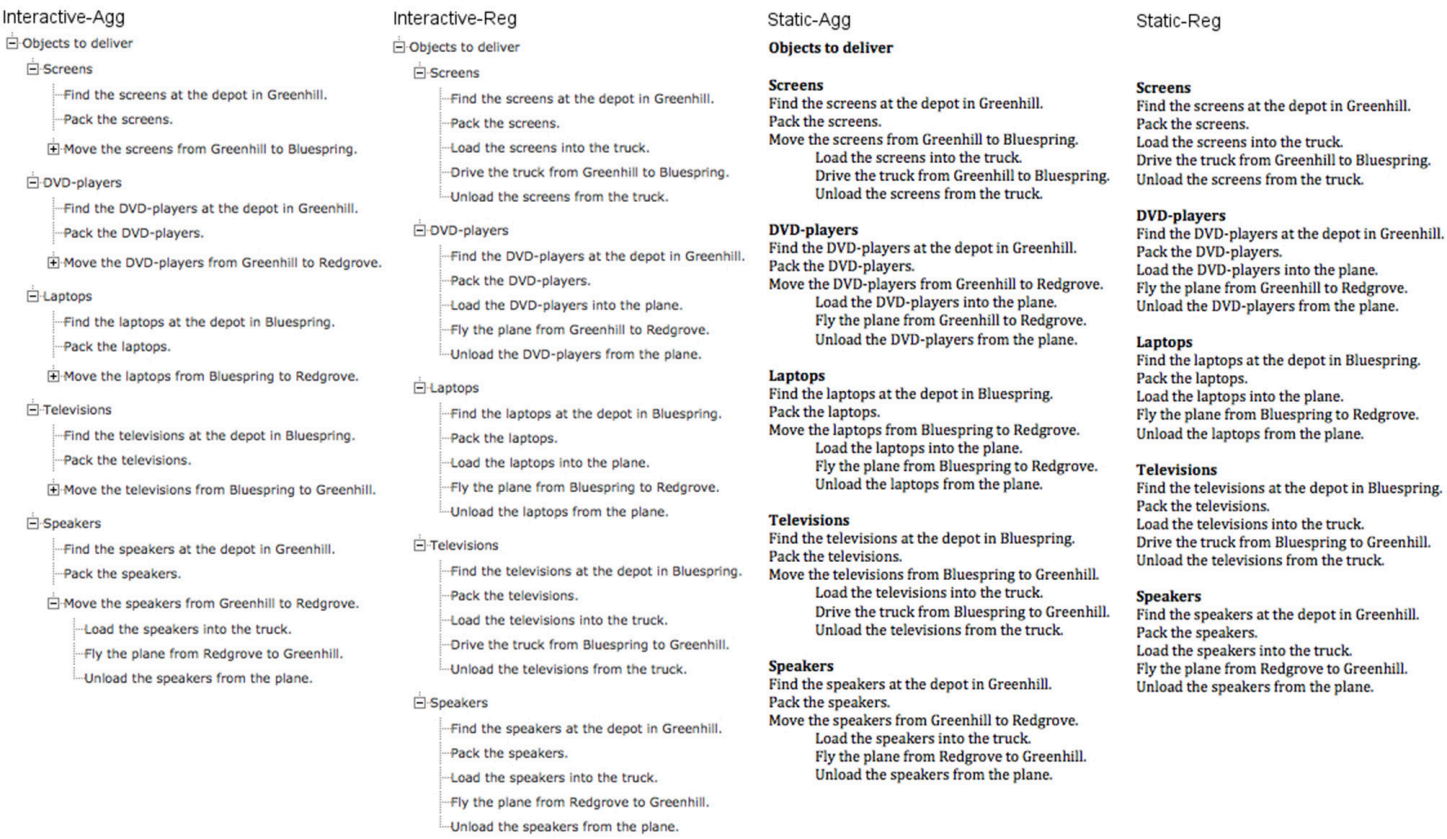
**The four conditions used in Experiment 1**.

We used four presentational options for plans: two variants of the static plan, and two of the interactive plan. Both variants differed on the level of aggregation (regular, or aggregated). Screenshots of the four plans used can be found in Figure 2. Participants always started with the most compact form of the interactive plans. The four variants are labeled as follows:

- Interactive-Agg. This is the (more) aggregated form of the interactive plan.
- Interactive-Reg. This is the regular form of the interactive plan.
- Static-Agg. This is the (more) aggregated form of the static plan.
- Static-Reg. This is the regular form of the static plan.

#### 4.3.2. Questions about the plan

Three different types of questions were asked: (i) questions about a step that is present in the plan, (ii) questions about a step that is not present in the plan but could have been, (iii) questions about ordering. In addition, each type of question was asked about steps that happened on a number of levels in the hierarchy in the plan e.g., does the step occur within the plan vs. does the step occur within a certain sub-plan? The questions asked were the following:

1. Do you drive the televisions to Greenhill?
2. Do you fly any of the planes to Redgrove?
3. Do you fly the screens to Bluespring?
4. Do you fly any of the planes to Bluespring?
5. What do you do first, fly a plane from Bluespring to Redgrove, or one from Greenhill to Redgrove?
6. What do you do first, pack speaker2 or fly a plane from Greenhill to Redgrove?

### 4.4. Design

The design was a between subjects design with 2 (Aggregated, Regular) ∗ 2 (Static, Interactive) variants.

### 4.5. Results

**H1. The number of correct answers in the secondary task will differ significantly between the representations**. Table 2 summarizes the performance in the four conditions. The difference is not significant between conditions. We note that the mean is low (ca 3/8), but comparable to previous findings (20% in Baddeley et al., 1975, 38% here).
**H1a. Scores will differ reliably across the levels of interactivity (interactive vs. static)**. Table 2 summarizes the number of words recalled across the two levels of interactivity. There was no reliable difference across the degree of interactivity.
**H1b. Scores will differ reliably across degree of aggregation (aggregated vs. regular)**. Table 2 summarizes the number of words recalled across the two levels of aggregation. There was no reliable difference across the degree of aggregation.
**H1c. The number of correct answers in the primary task will differ significantly between the representations**. There was no reliable difference across the four conditions.

**Table 2:** **Experiment 1, Mean (StD) in secondary task of number of words remembered correctly in the serial recall task out of maximum 8, in primary task of plan questions answered correctly out of maximum 6, and of digit span**.

		***Static-Agg***	***Static-Reg***	***Interactive-Agg***	***Interactive-Reg***	**All**
**Secondary task**	H1	2.86 (2.19)	3.45 (2.11)	2.23 (2.07)	3.09 (2.37)	2.90 (2.20)
	H1a	**Static**		**Interactive**		
		3.14 (2.15)		2.67 (2.25)		
	H1b	**Agg**		**Reg**		
		2.55 (2.13)		3.26 (2.24)		
**Primary task**	H2	5.27 (1.08)	5.20 (0.95)	4.50 (1.34)	4.65 (1.34)	4.90 (1.22)
**Digit span**		7.31 (1.25)	7.76 (1.20)	7.44 (0.92)	7.60 (1.30)	7.53 (1.15)

### 4.6. *Post-hoc*

We find large standard deviations in all conditions. In this section we use an analysis of variance (UNIANOVA), with digit span as a co-variate, to investigate whether differences in participants' working memories (measured as maximal digit span) may have influenced our findings comparing the different ways of presenting plans. Both analyses passed Levene's test of equality of error of variances.

**Digit span means**. Firstly we investigate the difference in digit span between conditions, summarized in Table 2. This mean does not vary significantly across conditions [*F*_(3)_ = 0.464, *p* = 0.708], but can still act as a co-variate. Next, we revisit the original hypotheses, while controlling for digit span.

**H1. The number of correct answers in the secondary task will differ significantly between the representations (controlling for working memory)**. We did not find a significant effect of condition [*F*_(3, 58)_ = 0.415, *p* = 0.743], digit span [*F*_(1, 58)_ = 2.31, *p* = 0.134], or interaction between condition and digit span [*F*_(3, 58)_ = 0.503, *p* = 0.682] on the number of correctly remembered words.
**H2. The number of correct answers in the primary task will differ significantly between the representations (controlling for working memory)**. We did not find a significant effect of condition [*F*_(3, 58)_ = 1.91, *p* = 0.137], digit span [*F*_(1, 58)_ = 0.65, *p* = 0.425] or interaction between condition and digit span [*F*_(3, 58)_ = 1.61, *p* = 0.196] on the number of correctly answered questions.

### 4.7. Discussion

We did not find that aggregation or interactivity helped task performance for plans (if anything, the trend was for them to diminish performance). The extra interaction may instead have created a certain degree of disorientation as has previously been found in hypertext (McDonald and Stevenson, 1996). That is, participants needed to interact with the plans before they could see all of the components. In the following sections we investigate an alternate hypothesis for why aggregation is not always helpful: that aggregation causes a certain degree of disorientation and lack of overview.

## 5. Experiment 3: highlighting emphasis only (lab)

In the last experiment we found no significant effect of emphasis (either highlighting or filtering) on cognitive load (measured in a dual-task paradigm). This experiment aimed to specifically evaluate the effectiveness of highlighting emphasis as a presentational choice. Since a system may sometimes incorrectly infer a goal, we also investigate the effect of such “unhelpful” emphasis as well, in relation to correct or “helpful” emphasis.

We investigate (a) whether emphasis as highlighting had an effect on errors and response times; and (b) if so, whether performance was improved by the mere presence of highlighting or if there was a difference when highlighting was for a different path through the plan than the one that a statement referred to (unhelpful highlighting). In the current experiment we compare the performance (response time and accuracy) for plans with no highlighting, with helpful and unhelpful highlighting.

This experiment helps us better to address RQ3: how we evaluate that the presentational choice is effective. For this reason, the evaluation protocol is different from the other experiments.

### 5.1. Experimental design

The experiment employs a full within-participants design, with all participants seeing all of the variants, in randomized order.

The independent variables are: (i) **Highlighting type**—whether the components of the plan that are highlighted constitute no highlighting, helpful highlighting, or unhelpful highlighting; and (ii) **true value**—whether the statement (e.g., “Give some grapes to Mary”) is true or false in relation to the plan. The dependent variables are: (a) **Response time**—the time taken to respond to the statement about the plan; and (b) **Errors**—the proportion of incorrect responses.

In the introduction screen participants were given the following instructions: “*On each screen you will be shown a plan and statement about the plan. For now, press any key to start a short practice session. This experiment studies different ways of presenting sequences of actions, or plans. You will be asked to press [true_key] if the statement is true and [false_key] if the statement is false.”*

**Figure 3:**
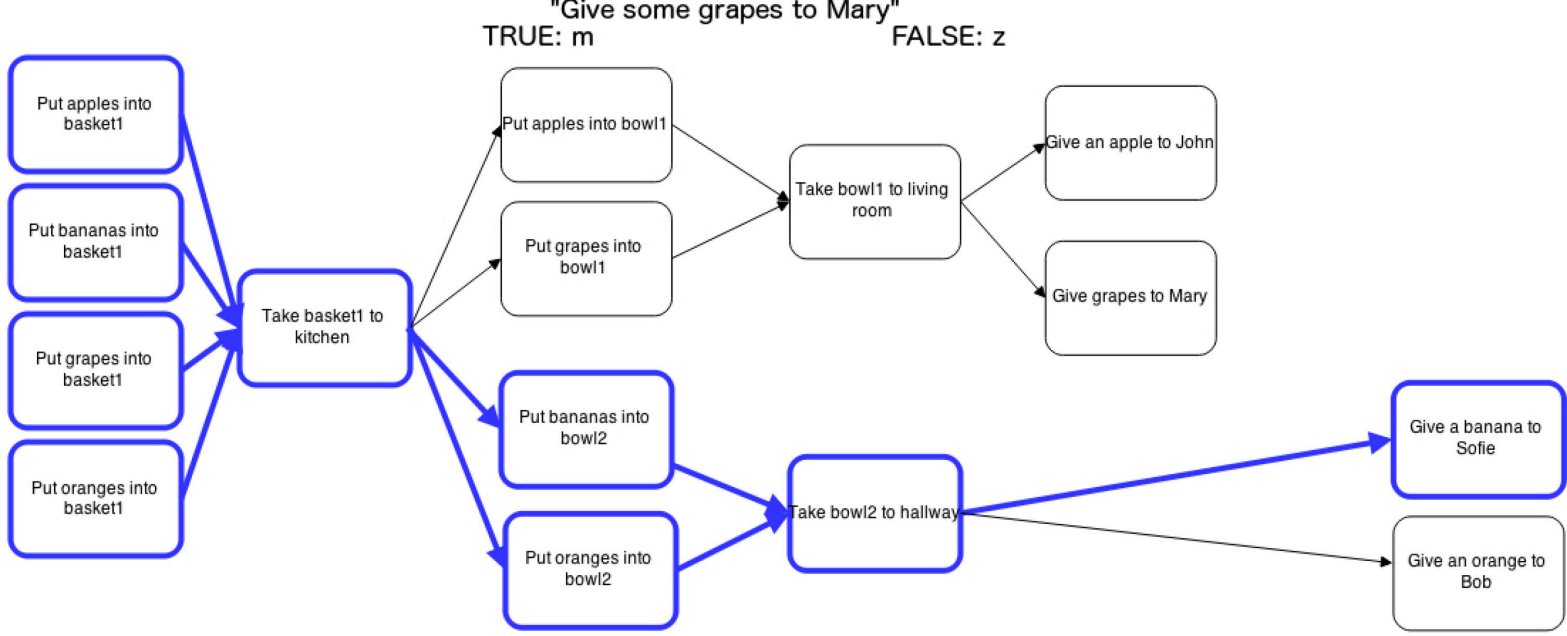
**Experiment 3, one experimental trial: plan and statement**. The highlighting is *unhelpful* for a statement about grapes, while the highlighting is helpful for bananas. The statement (“Give some grapes to Mary”) is *true* since the step with grapes nevertheless is present in the plan.

In each trial participants saw a statement and a plan (see Figure 3), and pressed a key to respond whether the statement was true or false for that plan. The keys for true/false were randomly assigned to either “m” or “z.” After each statement, participants were given quick feedback as a red or green dot with feedback text (either “correct” or “incorrect”) before going on to the next trial.

Participants first completed a practice session (6 trials) before going on to the experimental trials (144). In addition to the independent variables we also included 6 different categories of items (farm, groceries, sports, stationery, furniture (filler), tableware (filler)), with 4 items in each (e.g., apple, grape, banana and orange). The filler items were included to make participant less aware of the key dependent variables. This gave a total of 144 trials: 6 categories ∗ 4 items ∗ 3 types of highlighting ∗ 2 truth values. A break was inserted half way through to avoid participant fatigue.

### 5.2. Materials

#### 5.2.1. Plans

The plans were similar to the highlighting ones in Experiment 2, using border emphasis to indicate pre and post-conditions. All of them had the same shape as Figure 3, and thus balanced in terms of width and number of steps, with only the names of the tasks replaced. Recall that the categories used in the experimental trials were: farm, groceries, sports, stationery, furniture (filler), tableware (filler). For each trial and plan four objects were described, for example in the fruit category plans the following items were described: apple, pear, grapes, and banana. The range of domains was selected to minimize the effects of prior knowledge, and to ensure the generalizability of results.

#### 5.2.2. Statements

The statements used in the experiment had four properties: category (e.g., fruit), item (e.g., apple), and the type of highlighting they were associated with (e.g., helpful, unhelpful, no highlighting) a truth value for the statement (i.e., whether or not the statement is true according to the plan). Figure 3 gives an example of a statement for the fruit category. The plan is highlighted for bananas, but the statement is about grapes, so this is unhelpful highlighting. The statement and its truth value are *true*; this is in the plan, but the steps for bananas are not highlighted.

### 5.3. Hypotheses

H1: Helpful highlighting stimuli lead to faster response times than the no highlighting and unhelpful highlighting conditions.
H2: Helpful highlighting stimuli lead to fewer errors than the no highlighting and unhelpful highlighting conditions.
H3: True statements will lead to faster response times than false statements.
H4: True statements will lead to fewer errors than the false statements.

### 5.4. Results

The statistical analyses reported below were carried out in the mixed effects regression framework using the R package *lme4* (Bates et al., 2013). This method is well suited for studying repeated measures (several trials per participant); it also allows us to model individual variations between subjects as might be expected by variation in visual working memory (Conati and Merten, 2007). Jaeger (2008) and Baayen et al. (2008) describe the analysis method and its relationship to ANOVA. Items in the filler categories were excluded from analysis, as these were merely introduced to create variability in the stimuli and avoid participants guessing the key dependent variables.

#### 5.4.1. Participants

Participants were thirty-seven psychology undergraduate students, participating in a psychology experiment as part of their coursework. Data from two participants were removed because their average response times or error rates were more than 3 SDs away from the mean across participants.

**H1: Helpful highlighting stimuli lead to faster response times than the no highlighting and unhelpful highlighting conditions**. Table 3 summarizes the results, means are calculated by participant and response times were log normalized. The trend is for helpful highlighting to result in quicker response times than both unhelpful and no highlighting, as predicted by H1. Three models were built for complete two-way comparisons (see Table 4): helpful-unhelpful, no-helpful, no-unhelpful highlighting. There is a significant difference between helpful highlighting and the other conditions (*p* ≤ 0.01), but no significant difference between unhelpful and no highlighting^2^. *H1 is supported - helpful highlighting decreases response times.*
**H2: Helpful highlighting stimuli lead to fewer errors than the no highlighting and unhelpful highlighting conditions**. Table 3 also summarizes the mean error rates. Overall, the error rates are very low, with only 5–8% errors on average. There are most errors in the unhelpful condition. Three models were built for complete two-way comparisons (see Table 4): helpful-unhelpful, no-helpful highlighting, no-unhelpful. There is a significant difference between the helpful highlighting and the other two conditions (*p* ≤ 0.01), but not between the no and unhelpful highlighting conditions. *H2 is supported, relevant highlighting leads to fewer errors*.
**H3: True statements will lead to faster response times than false statements**. Table 3 summarizes the response times for true and false statements, with faster responses for true trials compared to false ones. In Table 4, we also see a significant difference in response times for each type of highlighting (*p* << 0.01). *H3 is supported: response times are reliably faster for true statements compared to false statements.*
**H4: True statements will lead to fewer errors than false statements**. Table 3 summarizes the error rates for true and false statements, with *more* errors for true statements. Table 4 shows that this difference is significant at *p* << 0.01 for all types of highlighting. Further, we found a significant interaction between type of highlighting and truth value in the comparison between unhelpful and no highlighting (*p* < 0.01). *H4 is not supported: true statements led to **more** errors compared to false statements*.

**Table 3:** **Experiment 3, response times as log(ms) and error rates by highlighting type and true value**.

		**Times**	**Times.sd**	**Errors**	**Errors.sd**
Highlighting type	Unhelpful	8.00	0.29	0.08	0.10
	No	8.02	0.27	0.05	0.07
	Helpful	7.86	0.33	0.05	0.08
True value	False	8.04	0.31	0.05	0.08
	True	7.88	0.27	0.07	0.09

**Table 4:** **Experiment 3, models for response times in log(ms) comparing highlighting conditions**.

			**Estimate**	**Std. Error**	***t*-value**	**Pr(>*t*)**
Response times	Unhelpful vs. helpful highlighting	(Intercept)	9.08	0.05	169.81	0.00
		Highlighting type	−0.14	0.04	−3.28	0.01
		true value	−0.17	0.03	−4.84	0.00
		Highlighting type^*^true value	−0.01	0.05	−0.27	0.79
	No vs. helpful highlighting	(Intercept)	9.08	0.05	190.68	0.00
		Highlighting type	−0.13	0.04	−3.16	0.01
		true value	−0.12	0.03	−3.55	0.00
		Highlighting type^*^true value	−0.06	0.05	−1.28	0.20
	No vs. unhelpful highlighting	(Intercept)	9.08	0.05	189.15	0.00
		Highlighting type	0.00	0.06	0.03	0.98
		true value	−0.12	0.03	−3.51	0.00
		Highlighting type^*^true value	−0.05	0.05	−0.98	0.33
Errors	Unhelpful vs. helpful highlighting	(Intercept)	9.08	0.05	169.81	0.00
		Highlighting type	−0.14	0.04	−3.28	0.01
		true value	−0.17	0.03	−4.84	0.00
		Highlighting type^*^true value	−0.01	0.05	−0.27	0.79
	No vs. helpful highlighting	(Intercept)	9.08	0.05	190.68	0.00
		Highlighting type	−0.13	0.04	−3.16	0.01
		true value	−0.12	0.03	−3.55	0.00
		Highlighting type^*^true value	−0.06	0.05	−1.28	0.20
	No vs. unhelpful highlighting	(Intercept)	1.94	0.02	121.22	0.00
		Highlighting type	0.00	0.01	0.29	0.77
		true value	−0.05	0.01	−3.46	0.00
		Highlighting type^*^true value	0.05	0.02	2.53	0.01

### 5.5. Discussion

As predicted we found the unhelpful highlighting increased errors and response times compared to helpful highlighting (or to even no highlighting at all). However, contrary to expectations (H4), we found that statements that are true led to *more errors* compared to false statements even if these evaluations were quicker. This suggests that participants “learn” to rely on the highlighting and anticipate the relevant parts of the plan to be highlighted, when in fact this is only true some of the time. This is further corroborated by a significant interaction between type of highlighting and truth value in the comparison between unhelpful and no highlighting. That is, participants made most errors when the statement was true, but the highlighting of the plan was unhelpful. If participants learned to rely on the highlighting this could also explain the longer response times for false statements, as participants may first look for confirmation in the highlighted parts of the plan before performing a more thorough search. A similar result was found when evaluating the effect of shortcuts in menu navigation on mobile phones—incorrect shortcuts led to more errors and slower performance (Bouzit et al., 2016).

## 6. Experiment 2: emphasis (mTurk)

In the first experiment (Section 4), we investigated the hypotheses that interaction and aggregation may benefit effectiveness for plans, but did not find that they helped task performance. In the following sections we investigate an alternate hypothesis for why aggregation is not always helpful: it causes a certain degree of disorientation and lack of overview. In the previous experiment participants needed to interact with the plans before they could see all of the components, and some of the questions addressed information at the deepest levels.

For this reason, in this Experiment we compare different ways of presenting an overview, while also keeping down the amount of information to avoid cognitive overload. Following the use of different colors in hypertext and hypermedia we also investigated the use of *emphasis* as a means to influence the presentational complexity. One variant we investigate is to show all the information, but filter it to only show required steps in a workflow following an algorithm originally proposed by Tintarev et al. (2014). Another variant we investigate is to retain all the information, but highlight the co-dependent steps. The highlighted variant allows users to retain an overview, whilst the filtered variant decreases the amount of visual working information. We also used color-coding of borders to distinguish between steps that have direct and indirect dependencies. These two variants (emphasis and filtering) were investigated as part of RQ2: how to present the plan visualization.

### 6.1. Procedure

The experiment ran on mTurk and followed the procedure outlined in Section 3.

The questions were shown one at a time rather than all at once, since the plan was large (1510x370 pixels) and it became difficult to show all the questions and the plan on the same screen. These plans used a visual language called Yet Another Workflow Language (YAWL), that visually represents when several steps need to be completed before moving on to the next step, and whether all (of multiple possible) next steps need to be completed. Additional support for pre- and post-conditions is given through border emphasis of steps. Participants can select an object, which triggers border emphasis of steps related to that object (involving the object directly or establishing pre-conditions). Color coding was used to distinguish between direct dependencies (steps describing a particular object) and indirect dependencies (other steps that needed to be completed but that did not directly involve the object).

Given the additional notation used in the plans in this experiment, an introductory screen with an example plan and illustrating questions was also supplied as training. This screen explained the notation, including highlighting colors and the visual language used. A brief primer to these can also be seen in the legend that was presented together with the plans (**Figure 5B**).

### 6.2. Design

The experiment followed a between subjects design with three variants: one for each type of presentational choice: highlighted, filtered and baseline.

### 6.3. Participants

Participants followed the same overall procedure as in Section 3, and were shown one variant of the plan in a between subjects design. Seventy-three participants completed the experiment. Six participants were removed from analysis, 5 for short completion durations (shorter than 1 SD from mean) and 1 due to a technical fault. 67 participants were used in analysis, with slightly more participants self-reporting as male than female (61% male, 39% female). The majority (66%) of participants self-reported to be aged 26–50, 21% said they were younger and 9% said they were older. The mean digit span was 6.24 (*SD* 1.16), and the mean time to complete was 12 min 3 s (*SD* 3 min 17 s).

### 6.4. Materials

#### 6.4.1. Presentational choices for plans

**Figure 4:**
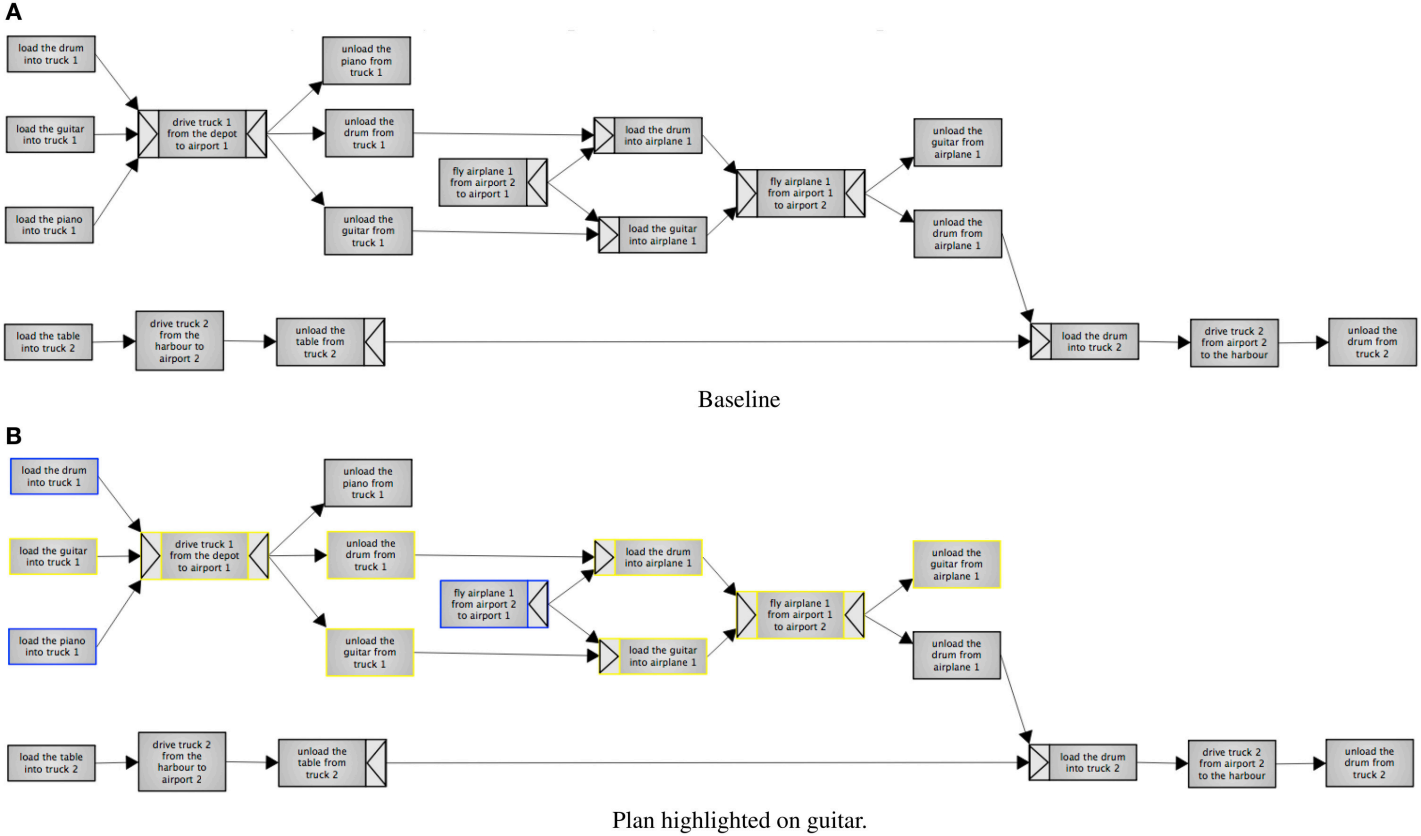
**Experiment 2, the baseline condition**. The baseline contains the full plan without any emphasis. Highlighting is on “guitar.” Steps with a blue border involve the guitar directly, whereas steps with yellow borders are dependencies. **(A)** Baseline. **(B)** Plan highlighted on guitar.

**Figure 5:**
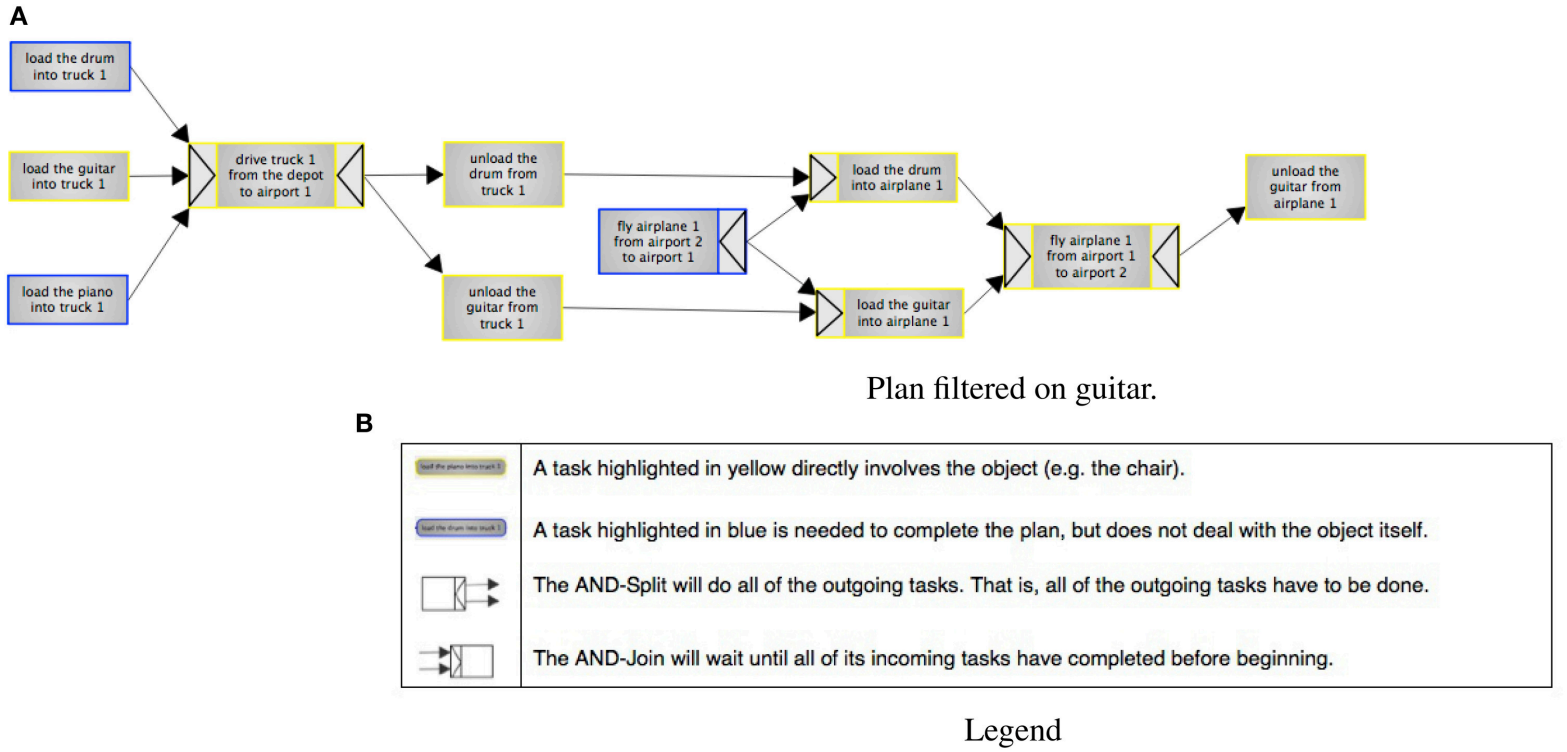
**Experiment 2, filtering on “guitar.”** Steps with a blue border involve the guitar directly, whereas steps with yellow borders are dependencies. The legend explains color coding, splits and joins. **(A)** Plan filtered on guitar. **(B)** Legend.

There were three variants of the same plan: highlighted, filtered and a baseline. Screenshots of the three variants can be seen in Figures 4A,B, 5A. The underlying plan for the three variants is taken from the International Planning Competition^3^. This plan is set in the delivery logistics domain, and describes how four objects (a truck, a piano, a table and a drum^4^) are delivered to different locations (cities and airports). The plan also contains a number of resources (trucks and airplanes). The plans are the output of a system that generates the graphs using the YAWL format from PDDL format. The system uses an algorithm introduced in (Tintarev et al., 2014) to select which steps to filter and highlight. The algorithm selects all the steps an item is directly involved in, as well as any dependencies that may be required to complete the plan. By dependencies we mean pre-conditions, or steps that are required before the main tasks can begin.

In the highlighted variant, participants select one out of four items (a piano, table, drum or guitar) to focus on. There was also a button for resetting the highlighting. This variant shows the whole plan, but highlights the steps selected by the algorithm. Steps that an item is directly involved in are highlighted in yellow, while dependencies are highlighted in blue. The filtering variant has the same highlighting and selection options, but also hides any steps that are not highlighted. The baseline is non-interactive, and simply displays the full plan.

Dependencies in YAWL can additionally be defined in terms of AND Joins and Splits, and are represented as arrows on the outside of edges. The AND-Split means that all the outgoing steps have to be done (unlike e.g., OR, where a choice can be made). The AND-Join will wait until all its incoming tasks have been completed before beginning.

The color-coding, joins and splits were all explained in a legend that was included both in the introduction and alongside the plans when participants were answering the questions. The included legend is shown in Figure 5B.

Participants were asked questions about ordering, dependency between items, overview questions and strategic questions. These questions were based on an adaptation of the three-level model of situational awareness (Endsley and Jones., 2012). The ordering questions look at the ability to correctly perceive information (level one). Dependencies and overview questions look at the ability to comprehend the situation (level two). The strategic questions look at projecting the situation into the future (level three). These are the questions asked:

- **Order questions**:
  - ∙ Can you unload the guitar from truck1 before you load the drum into truck1? (yes/no)
  - ∙ Can you drive truck2 to the harbor before you unload the piano from truck1? (yes/no)
  - ∙ Can you drive truck2 to airport2 before flying airplane1 the first time? (yes/no)
- **Dependency questions**:
  - ∙ The table has been unloaded from Truck2, and airplane1 has flown to airport2. What else needs to happen before you can drive truck2? (unload the guitar/unload the drum)
  - ∙ Airplane1 has arrived to Airport1. You have unloaded everything from truck1. Is it at all possible for you to deliver the guitar to its final destination? (yes/no)
  - ∙ Truck2 has a technical fault and cannot drive. Is it at all possible for you to deliver the drum to its final destination? (yes/no)
- **Overview questions**:
  - ∙ Does the guitar get delivered to the same place as the table? (yes/no)
  - ∙ Does the guitar get delivered to the same place as the drum? (yes/no)
- **Strategic questions**:
  - ∙ How many objects are being delivered? (three/four)
  - ∙ Which vehicle will cause the FEWEST number of objects to be delivered if it breaks down? (airplane1, truck1, truck2)

### 6.5. Results

**H1. The number of correct answers in the secondary task will differ significantly between the representations**. Table 5 summaries the number of correctly remembered words. For all conditions, the number of words is a lot lower than in Experiment 1 (*m* = 2.90), suggesting that this task may have been more cognitively demanding. There is no significant difference between conditions [ANOVA, *F*_(2)_ = 2.06, *p* = 0.36].
**H2. The number of correct answers in the primary task will differ significantly between the representations**. Table 5 summarizes the number of plan questions answered correctly. We see that in all conditions, the number of correctly answered questions is low, with means around 6 (out of 10). The differences were not significant [ANOVA, *F*_(2)_ = 5.09, *p* = 0.78].

**Table 5:** **Experiment 2, Mean (StD) in secondary task of number of words remembered correctly in the serial recall task out of maximum 8, in primary task of plan questions answered correctly out of maximum 10, digit span performance, and estimated marginal means (StD) for primary task of plan questions answered correctly out of maximum 10 with covariate digit span evaluated at 6.24**.

	**Baseline**	**Filter**	**Highlight**	**All**
Secondary task	2.41 (2.34)	1.81 (2.32)	2.29 (1.90)	2.18 (2.17)
Primary task	5.73 (1.24)	5.81 (1.40)	6.42 (2.15)	6.00 (1.67)
Digit span	6.55 (1.06)	6.38 (1.07)	5.83 (1.24)	6.24 (1.16)
Primary task controlling for digit span	5.23 (0.36)	5.67 (0.36)	6.55 (0.35)	–

### 6.6. *Post-Hoc* analysis

None of the findings in this experiment were statistically significant. However, the large standard deviations suggest that a within condition variation may have been larger than the effect of the between condition manipulation. We apply an analysis of variance (UNIANOVA)^5^, with digit span as a co-variate, to investigate whether differences in participants' working memories (measured as maximal digit span) may have influenced these findings. Both analyses passed Leven's test of equality of error of variances. We tested for main effects of digit span, condition, and the interaction between them.

**Digit span means**. Firstly we investigate the difference in digit span between conditions, summarized in Table 5. This mean does not vary significantly across conditions [*F*_(2)_ = 2.52, *p* = 0.09], but can still act as a co-variate. Next, we revisit the original hypotheses, while controlling for digit span.

**H1. The number of correct answers in the secondary task (wordlist) will differ significantly across the three ways of presenting plans (controlling for working memory)**. We did not find a significant effect of condition [*F*_(2)_ = 0.57, *p* = 0.57], digit span [*F*_(1)_ = 2.67, *p* = 0.11] or interaction between them [*F*_(2)_ = 0.42, *p* = 0.66] on the word list recall.
**H2. The number of correct answers in the primary task (plan questions) will differ significantly across the three ways of presenting plan. (controlling for working memory)**

The mean for digit span does not vary significantly across conditions [*F*_(1)_ = 1.31, *p* = 0.257], but can still act a co-variate influencing task performance. Indeed, controlling for digit span we found a significant effect of condition [*F*_(2)_ = 4.92, *p* = 0.01] on task performance. The interaction between condition and digit span (DS) was also significant [*F*_(2)_ = 3.59, *p* = 0.03]. Table 5 summarizes the means with the co-variate factored in.

Using simple contrasts we found a significant difference between highlighting and the baseline conditions, but not for any of the other pair-wise comparisons (*p* ≤ 0.01). For pair-wise comparisons, Table 6 summarizes the effect sizes of the condition, digit span and the interaction between them. The negative β values for the interactions Filtering^*^DS and Highlighting^*^DS describe a negative relationship between digit span and these two conditions. Only the interaction with highlighting is significant however. This suggests that while highlighting can be effective, it is less effective for participants with poorer memory.

**Table 6:** **Experiment 2, Effect sizes for main effects of condition, digit span (DS), and the interaction between them**.

**Parameter**	**β**	***p***
Filtering	4.60	0.14
Highlighting	8.44	**0.003**
Baseline	0	.
DS	0.80	**0.018**
Filtering^*^DS	−0.64	0.18
Highlighting^*^DS	−1.14	**0.010**
Baseline^*^DS	0	.

Values significant at p < 0.05 are in bold.

## 7. Experiment 4: emphasis (lab)

In Experiments 1 and 2, we saw that there was a high variation of performance for both primary and secondary tasks between participants. These variations can be due to a large number of factors, but generally these findings suggest that factors other than emphasis (i.e., highlighting and filtering) affected performance. In the last experiment (Experiment 4) we ruled out a possible explanation for this: that the emphasis was not helpful at all (and the variation was general noise). We found that the sort of emphasis actually decreased errors and decreased reaction times. Experiment 4 was however run in the lab and we wished to investigate an alternate hypothesis: if the high variance for Experiments 1 and 2 was an artifact of running experiments on mTurk. In this section we therefore introduce an experiment that investigates the extent of within-participant variation in controlled lab conditions.

This version of the experiment also considers several issues identified in two sets of co-discovery tasks with 6 pairs of participants, aimed to identify any general usability issues. The main findings in the co-discovery sessions were that the filtering and highlighting were not sufficiently visible and not sufficiently useful: even when the pairs found the functionality it was hardly used. Additionally, several aspects of the interface were difficult to interpret. These included the shapes that indicated that all actions were required (AND) to either start or complete a sequence. Participants also found it difficult that there was both blue and yellow highlighting. This study therefore introduces the use of a training phase to increase the visibility of the emphasis, and a more simplified and explicit notation for dependencies.

This experiment continues answering the question posed in Experiment 2, if emphasis and filtering are good ways to present plans (RQ2). It also addresses potential methodological issues with mTurk and procedural issues identified in a co-discover task, providing a better understanding of the answer to RQ3 (how do we evaluate).

### 7.1. Procedure

Aside from being lab-based, the procedure of this experiment is very similar to Experiment 3. Based on the findings of the co-discovery task an extended training phase was added to help users become familiar with the notation. Similarly, given the comments on the complexity of the notation, the plans were also modified (see the Materials section). We were also interested to see if visual memory influences performance and used a visual working memory test instead of digit span^6^.

### 7.2. Participants

Sixty-seven participants were recruited from university mailing lists and message boards, and included both staff and students across all disciplines and departments. These were self reported fluent in English (although not necessarily native speakers). Of these 44 were female (65.7%) and 23 male (34.4%). 59.7% of participants were aged 18–25, 29.9% aged 26–35, 3 aged 36–45, 3 aged 46–56, and 1 aged 57+. The average visual working memory was 5.33 (std 0.73). Average completion time was 15.58 min (std 5.22). Participants were paid £5 for an estimated completion time of 30 min.

### 7.3. Materials

#### 7.3.1. Presentational choices for plans

**Figure 6:**
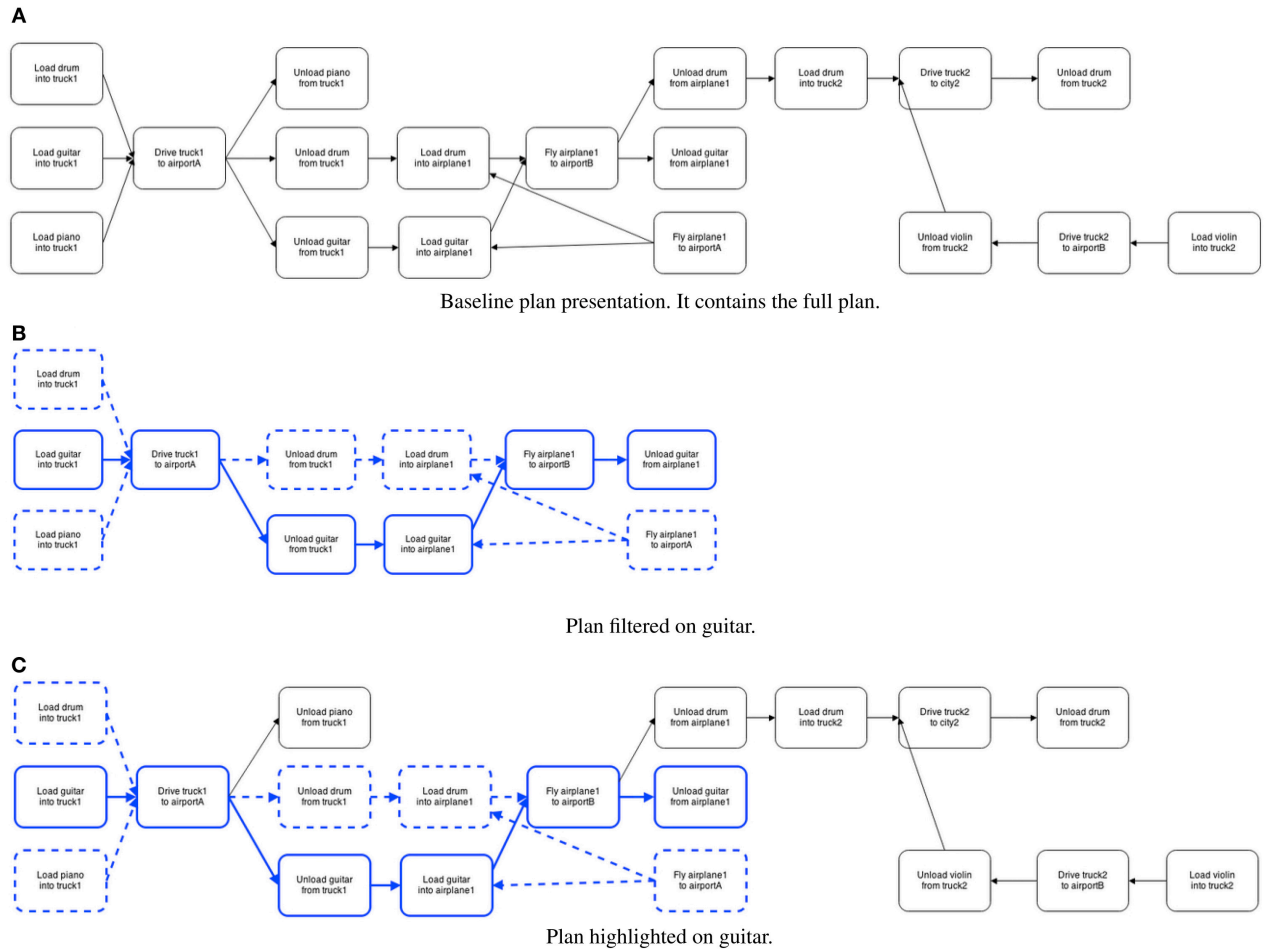
**Experiment 4, the three conditions**. Baseline, and also highlighting vs. filtering of the plan on “guitar.” Emphasis is marked by a thicker and colored (blue) border. Dependencies are indicated by dotted lines. **(A)** Baseline plan presentation. It contains the full plan. **(B)** Plan filtered on guitar. **(C)** Plan highlighted on guitar.

The plan notation was simplified. The shapes that indicated that all actions were required (AND) to either start or complete a sequence were removed. We limited the highlighting to one color (blue), but included another button for dependencies (which were indicated by dotted lines in the same color). The resulting plans can be seen in Figures 6A–C. To make the emphasis more useful, we also increased the types of filtering allowed (e.g., also by vehicle and by location). Users could select how the emphasis was applied by selecting buttons placed above the plan. There were buttons for each of the objects, vehicles, and locations. To the right, after a gap, there was also a button called “*Dependency.”* This indicates the user wants to see the pre-requisites for completing the tasks related to the selected thing (e.g., guitar, or airportA) rather than only steps that involve the thing directly. Furthest to the right, participants could also select “*No filter”* to clear any emphasis.

### 7.4. Results

**H1. The number of correct answers in the secondary task (wordlist) will differ significantly between the representations**. Table 7 summarizes the number of correctly remembered words. For all conditions, the number of words remembered is similar to the mTurk experiment comparing highlighting and filtering (c.f. Experiment 2). As expected from the means, there is no significant difference between conditions [ANOVA, *F*_(2)_ = 0.001, *p* = 0.999].
**H2: The number of correct answers in the primary task (plan questions) will differ significantly between the representations**. Table 7 summarizes the means of correctly answered questions. As in the mTurk experiment comparing highlighting and filtering (Experiment 2), there is no significant difference between conditions [ANOVA, *F*_(2)_ = 0.45, *p* = 0.60].

**Table 7:** **Experiment 4, Mean (Std), in secondary task of number of words remembered correctly in serial recall task out of maximum 8, in primary task of plan questions answered correctly out of maximum 10, and of visual memory**.

	**Baseline**	**Filter**	**Highlight**	**All**
Secondary task	2.35 (2.29)	2.35 (2.27)	2.32 (1.89)	2.34 (2.13)
Primary task	5.87 (1.25)	6.05 (1.46)	6.23 (1.07)	6.04 (1.26)
Visual memory	5.46 (0.89)	5.27 (0.55)	5.25 (0.72)	5.33 (0.73)

### 7.5. *Post-hoc*

The results in the lab study largely replicate the large variation between participants that we found on mTurk. This suggests that variation within participants may be due to more than using mTurk.

In Experiment 2, we found that working memory, measured as digit span, influenced the number of correctly answered questions about a plan. We applied an analysis of variance (UNIANOVA) to investigate whether differences in participants' *visual working memory* as measured in the Corsi test may have influenced our findings comparing the different ways of presenting plans. Both analyses passed Leven's test of equality of error of variances. We tested for main effects of visual working memory, condition, and the interaction between them.

**Visual working memory means**. Firstly, we investigate the difference in visual working memory between conditions, summarized in Table 7. This mean does not vary significantly across conditions [*F*_(2)_ = 0.536, *p* = 0.588], but can still act a co-variate influencing task performance. Next, we revisit the original hypotheses, while controlling for visual working memory.

**H1. The number of correct answers in the secondary task (wordlist) will differ significantly across the three ways of presenting plans (controlling for visual working memory)**. We did not find a significant effect of condition [*F*_(2)_ = 1.206, *p* = 0.306], visual working memory [*F*_(1)_ = 0.216, *p* = 0.644] or interaction between condition and visual working memory [*F*_(2)_ = 1.222, *p* = 0.302] on the word list recall.
**H2. The number of correct answers in the primary task (plan questions) will differ significantly across the three ways of presenting plans (controlling for visual working memory)**. We found that working memory had a significant effect on the number of correctly answered plan questions [*F*_(1)_ = 4.719, *p* <0.05]. However, there was no significant interaction between the conditions and working memory [*F*_(2)_ = 0.10, *p* = 0.904].

For pair-wise comparisons, Table 8 summarizes the effect sizes of the condition, visual working memory and the interaction between them. These results suggests that individual differences in visual working memory were better predictors for the variance in task performance, than the information presentational choices.

**Table 8:** **Experiment 4, effect sizes for main effects of condition, visual working memory, and the interaction between them**.

**Parameter**	**β**	***p***
Filtering	−1.462	0.572
Highlighting	−0.148	0.964
Baseline	0	.
WM	0.437	0.249
Filtering^*^WM	0.186	0.699
Highlighting^*^WM	−0.008	0.989
Baseline^*^WM	0	.

## 8. Summary and discussion

We first studied the effect of interactivity and aggregation on task performance (Experiment 1). We were not able to find a benefit for making plans interactive or using aggregation. These choices did not result in any difference in performance *between* conditions on the secondary task.

In the remainder of the paper, we rule out a number of possible explanations for why interactivity did not improve performance. First we considered whether hiding parts of a plan can cause disorientation and influence performance. In an initial comparison of emphasis and filtering (Experiment 2), we saw that working memory (digit span) influenced the number of correctly answered questions about a plan. It also interacted with highlighting—how good border emphasis was depended on individual differences in working memory. (No such interaction was found for the filtering condition which we thought might be the most disorienting). Participants were given the same wordlist in Experiment 1 ($\bar{m}\approx$ 3) and Experiment 2 ($\bar{m}\approx$ 2), but the number of words remembered was on average lower in Experiment 2. One possible explanation is that participants were under heavier cognitive load because the plans in Experiment 2 were more complex.

In Experiment 3, investigated the possibility that border emphasis is simply not effective. In this more controlled experiment we found that border emphasis decreased reaction times and error rates. This suggests that our choice of visual representation actually does help users, as long as we control for individual variation.

We also considered whether there were any general user interface issues given that Experiment 3 did not find significant differences between conditions (unless we considered individual differences in memory). In a co-discovery evaluation (described in the beginning of Section 7) we identified that highlighting and filtering were not being used by all users. As a result we increased the visibility of this functionality using a training phase, and by increasing the types of filtering available (e.g., by location and resource). This follows the strategy recommended by Gould et al. (2015b) who found no differences between results in the lab and mTurk on a number of dimensions (e.g., number of errors or durations), but highlights the importance of properly introducing mTurk participants to experimental procedures to avoid misunderstandings.

These issues were considered in the final experiment (Experiment 4). In this experiment, we investigated if the high variance in results was an artifact of running experiments on mTurk. In this controlled lab study we found a large variance between conditions, replicating the findings of the earlier experiments. We also found similar results for visual working memory as we did for working memory measured as a digit span: the individual differences in *visual working memory* had a large impact on task performance. This suggests that the variance between participants is not an artifact of conducting studies on mTurk.

### 8.1. Limitations

Firstly, one could question the validity of studies performed on mTurk compared to those conducted in a lab. For example, Gould et al. (2015a) found that a small, but notable, number of participants interleave activities while conducting an experimental HIT with a lock-out (i.e., an inbuilt delay for progressing). In our experiments, where we were measuring performance in a dual-task this is likely to greatly influence our results. However, the same authors also found that with appropriate selection and data-cleaning there is no significant difference in participant performance along a number of dimensions (Gould et al., 2015b).

So, while additional interruptions to mTurk workers may have explained some of the variance in our results, our control measures (in addition to mTurk approval ratings) with regard to minimal performance on a memory test, maximum task duration, and removing participants with very short duration times, should have mitigated this effect. Indeed, the similar variation between performance in participants also in our lab studies, suggests that our selection has been effective. This is also corroborated by completion times. Completion times in Experiments 1 and 2 on mTurk took an average of about 12 min, and Experiment 4 in the lab took closer to 16 min. These shorter durations for mTurk workers suggest that they were unlikely to be inter-leaving tasks (and taking longer).

In contrast, the shorter times suggest that mTurk workers may have taken the task less seriously than participants in the lab. We note however, that while slightly shorter, these times are not *greatly* divergent from the time taken in the lab. Our results are also supported by a wider body of studies confirming the comparable quality of results from mTurk studies when carefully designed (Dandurand et al., 2008; Kittur et al., 2008; Heer and Bostock, 2010; Paolacci et al., 2010; Komarov et al., 2013; Gould et al., 2015b).

Secondly, there may be some concerns about the ecological validity of second task (word list recall). It is unlikely that an operator will have to remember a sequence of (to each other) unrelated items while performing a task or making decisions. However, it is very likely, that they will be interrupted during the execution of one task, to start the execution of another. This additional cognitive load will affect operators to varying extents depending on their working memories, and previous research has shown that presentation of information can benefit from tailoring to working memory. The wordlist was chosen, since we have an empirical baseline for how difficult it is to recall without additional cognitive load (Baddeley et al., 1975). It would be difficult to find another task for which the load is as well controlled and understood. The wordlist also places a different type of load on memory, i.e., on verbal working memory rather than visual working memory. This avoided the potentially confounding factor of overloading the same modality when measuring task performance.

### 8.2. Future work

Emphasis via filtering and emphasis on objects, resources and locations are only some ways to tailor plan presentations to a user. A user's knowledge and their capabilities to perform actions should be taken into account when deciding on a plan presentation that is suitable. Our future work will study whether tailoring information presentation visually for degree of expertise is useful, and whether it creates an over-reliance on the presented information (as we found for the highlighting in Experiment 4). It will also study other ways of tailoring the information w.r.t. laying out the plan—e.g., temporal vs. geographical layouts. Given the strong findings for the influence of individual differences in the experiments described in this paper, these future studies will apply both repeated measures and within subjects methodologies: each participant will see several presentational choices and several concrete examples for each presentational choice.

While this work has investigated some presentational choices, there are many other open questions with regard to how to best present plans. Gestalt theory suggests that a plan may be viewed as a unified whole or an organization of groups of elements (Wertheimer, 1923). In this paper we considered a single layout which described a logical ordering (left-to-right), and geographic location (vertical “lanes”). Our future work will consider the Prägnanz principles (e.g., similarity, proximity and continuity) interact with different choices for layout. Our next studies will explore other visual representations of graphs with different types of inherent complexity such as cycles (re-occurring parts of the plan), and concurrency (events that happen in parallel). Also, given the effect of individual differences in working memory on *effectiveness*, another avenue of research we plan to pursue is an investigation of whether similar effects can be found for *memorability* of information w.r.t. both short- and long-term memory.

## 9. Conclusion

This paper aims to address three research questions: *(RQ1) whether individual user differences in working memory should be considered when choosing how to present visualizations; (RQ2) how to present the visualization to support effective decision making and processing; and (RQ3) how to evaluate the effectiveness of presentational choices.* To address these questions, it describes four experiments evaluating the effectiveness of different ways of visually representing a plan, summarized in Table 9. These experiments consider individual differences in working memory as both digital span and visual working memory **(RQ1)**. With regards to presentational choices we consider a range of presentational choices such as layout, degree of interactivity, aggregation and emphasis **(RQ2)**. These choices are evaluated primarily using a dual-task paradigm to simulate task interleaving **(RQ3)**. Two crowd-sourced experiments on mTurk are complemented with methods such as co-discovery and repeat measures in more controlled lab studies.

**Table 9:** **Overview of experiments and results**.

**Exp**.	**Setting**	**Result**
1	mTurk	Tested the effect of aggregation and interactivity for larger (125 steps) plans. No significant difference for number of correctly answered questions for interactive plans compared to static plans. Controlling for WM did not influence result on primary or secondary task performance.
2	mTurk	WM influenced the number of correctly answered questions about a plan. It also interacted with emphasis (highlighting).
3	Lab	Helpful emphasis in a plan improved the number of correct responses and decreased the number of errors. Unhelpful emphasis led to more incorrect responses.
4	Lab	Individual differences in *visual* WM were better predictors for the variance in task performance than emphasis (filtering and highlighting).

In closing, we recommend that systems applying visual representations of plans consider differences in working memory (RQ1), since in some cases the representation may be another feature that taxes users cognitively. As Conati et al. (2014) found an effect of working memory on performance for different visual layouts, so did we find an effect of working memory on performance due to emphasis (RQ2: how to present). This suggests that there are cognitive motivations for personalizing visual information presentation, as studied by Mutlu et al. (2015).

Our results also stress the importance of repeated measures studies (such as Experiment 3) (RQ3: how to evaluate). While repeated measures experiments create a greater burden on the creation of experimental materials, and testing time, they allow to control for individual variation in ways that other experimental set-ups do not. Our results also demonstrate the value of multiple evaluation measures and in particular a dual task methodology for evaluating the effectiveness of visual presentational choices for plans (RQ3).

## Ethics statement

Ethics committee for the College of Physical Sciences, University of Aberdeen. For studies on Amazon Mechanical Turk, participation was voluntary and participants were shown an information sheet before starting. In lab studies, participants were given verbal instructions in addition to signing written consent forms after seeing an information sheet. For all experiments, participants could withdraw at any time without penalty. All participants were also told that the data would be anonymized and used for research purposes only. No additional considerations are likely to be relevant.

## Author contributions

Substantial contributions to the conception or design of the work (NT, JM); acquisition and analysis of data for the work (NT, JM); Revising the work critically for important intellectual content (NT, JM); Final approval of the version to be published (NT, JM).

### Conflict of interest statement

The authors declare that the research was conducted in the absence of any commercial or financial relationships that could be construed as a potential conflict of interest.

## Footnotes

^1^The average person has a memory span of between 5 to 9 digits Miller (1956).

^2^Significance levels given using R package lmerTest, http://cran.r-project.org/web/packages/lmerTest/index.html, retrieved April 2015

^3^http://ipc.icaps-conference.org/, retrieved April 2015.

^4^In the original plan they are given names such as “obj11,” and we have mapped these to more human intelligible labels.

^5^The results were also confirmed using a MANOVA.

^6^Having a measure of both would have been ideal, however the expected fatigue of participants who did both memory tests would likely influence performance on primary and secondary task.
